# Genetics Is of the Essence to Face NAFLD

**DOI:** 10.3390/biomedicines9101359

**Published:** 2021-09-30

**Authors:** Marica Meroni, Miriam Longo, Giada Tria, Paola Dongiovanni

**Affiliations:** 1 General Medicine and Metabolic Diseases, Fondazione IRCCS Ca’ Granda Ospedale Maggiore Policlinico, Pad. Granelli, Via F Sforza 35, 20122 Milan, Italy; maricameroni11@gmail.com (M.M.); longo.miriam92@gmail.com (M.L.); triagiada1999@libero.it (G.T.); 2 Department of Clinical Sciences and Community Health, Università Degli Studi di Milano, 20122 Milano, Italy

**Keywords:** NAFLD, heritability, personalized medicine, lipid handling, polygenic risk scores

## Abstract

Nonalcoholic fatty liver disease (NAFLD) is the commonest cause of chronic liver disease worldwide. It is closely related to obesity, insulin resistance (IR) and dyslipidemia so much so it is considered the hepatic manifestation of the Metabolic Syndrome. The NAFLD spectrum extends from simple steatosis to nonalcoholic steatohepatitis (NASH), a clinical condition which may progress up to fibrosis, cirrhosis and hepatocellular carcinoma (HCC). NAFLD is a complex disease whose pathogenesis is shaped by both environmental and genetic factors. In the last two decades, several heritable modifications in genes influencing hepatic lipid remodeling, and mitochondrial oxidative status have been emerged as predictors of progressive hepatic damage. Among them, the patatin-like phospholipase domain-containing 3 (PNPLA3) p.I148M, the Transmembrane 6 superfamily member 2 (TM6SF2) p.E167K and the rs641738 membrane bound-o-acyltransferase domain-containing 7 (MBOAT7) polymorphisms are considered the most robust modifiers of NAFLD. However, a forefront frontier in the study of NAFLD heritability is to postulate score-based strategy, building polygenic risk scores (PRS), which aggregate the most relevant genetic determinants of NAFLD and biochemical parameters, with the purpose to foresee patients with greater risk of severe NAFLD, guaranteeing the most highly predictive value, the best diagnostic accuracy and the more precise individualized therapy.

## 1. Introduction

Nonalcoholic fatty liver disease (NAFLD) is the most frequent chronic liver disorder of the 21st century, affecting at least one third of the general population [1,2,3]. Due to its epidemic proportion, NAFLD constitutes a huge socio-economic and health issue [4] and it is predicted to become the leading cause of hepatocellular carcinoma (HCC) and the main indication of liver transplantation by 2030 [5]. NAFLD is defined by ectopic fat deposition exceeding 5% of liver weight, in absence of alcohol consumption. It embraces a variable phenotypic rainbow of hepatic abnormalities, spreading from uncomplicated steatosis to its progressive form, nonalcoholic steatohepatitis (NASH), characterized by lobular inflammation, hepatocyte ballooning degeneration and fibrosis. NASH may then evolve towards end-stage liver injuries, such as cirrhosis and HCC [6,7].

NAFLD is epidemiologically related to obesity, insulin resistance (IR) and atherogenic dyslipidemia so much so it is considered the hepatic manifestation of Metabolic Syndrome [8,9]. Hence, according to a recent international consensus, the nomenclature of NAFLD has been updated from NAFLD to metabolic dysfunction-associated fatty liver disease (MAFLD), to better outline patients in which hepatic steatosis occurs in the presence of obesity or type 2 diabetes (T2D) or metabolic abnormalities [10].

However, NAFLD has an intricate pathogenesis and 50–70% of the individual susceptibility to develop the disease as well as its phenotypic variability are attributable to inherited risk factors [11]. The most robust genetic predictors of NAFLD are single nucleotide polymorphisms (SNPs) in genes regulating hepatic lipid turn-over, reshaping and dismissal, among which patatin-like phospholipase domain-containing 3 (*PNPLA3*), transmembrane 6 superfamily member 2 (*TM6SF2*), membrane bound o-acyltransferase domain-containing 7 (*MBOAT7*) and Glucokinase regulator (*GCKR*) [11]. Even more, along with the heritable variations, gene-environment interactions may also explain the discrepancies in NAFLD phenotypic variability, possibly amplifying the effect due to individual sequence variations [12,13]. For instance, the associations between common variants and NAFLD may be unmasked by the increased adiposity, thus enhancing the genetic risk [14]. In addition, among the different actors who play a role in NAFLD pathophysiology, a new point of view is constituted by intestinal dysbiosis, enhanced intestinal permeability and microbial harmful by-products [15,16].

Nowadays, liver biopsy remains the gold standard procedure for diagnosis of NAFLD and no therapeutic consensus exists for its treatment [9,17]. However, the combination of inherited factors and dynamic clinical parameters, which can be influenced by lifestyle and pharmacological interventions, may be effective to identify reliable score-based approaches aimed to predict liver damage and to tailor therapeutic options [9,17].

In summary, this review aimed to offer a systematic overview of the genetic risk factors currently known to be related to NAFLD pathogenesis, particularly addressing to their assessment to ameliorate non-invasive disease diagnosis and to personalize the clinical management of the disease.

## 2. Historical Overture to Discover the Link between Genetics and NAFLD

In the last decade, it has been broadly elucidated that obesity and IR are the leading risk factors for NAFLD. However, at equal body mass index (BMI), there is a widespread variability in the clinical manifestation of NAFLD, supporting the notion that other jeopardizing factors may be engaged into fatty liver onset and progression. Indeed, familial, twin and epidemiological studies pinpoint that both steatosis and fibrosis have a huge inherited component [18,19].

The first robust evidence regarding NAFLD hereditability has been provided by Struben et al. [20], who studied the familial pattern distribution of cryptogenic cirrhosis in 18 members of 8 kindreds, containing 2 or more afflicted members. These authors revealed that the coexistence of NASH with or without cirrhosis within kindreds suggests a common etiology of these disorders, possibly caused by the shared genetic background and by the elevated frequency of obesity and T2D in these families. Then, large population-based studies more precisely outlined the magnitude of NAFLD predisposition due to genetics. Indeed, Speliotes and colleagues [21] attested the hereditability of hepatic steatosis at 26−27% in a population-based consortia including 6629 subjects of European descent. This estimate has been confirmed by Wagenknecht et al. in 795 Hispanic American and 347 African-American adults who participated to the Insulin Resistance Atherosclerosis Study (IRAS) Family Study [22].

More in detail, in a familial aggregation study, Schwimmer et al. revealed that family members of overweight children with biopsy-proven NAFLD had an increased predisposition to develop hepatic steatosis compared to obese children without NAFLD [23]. Thus, a familial NASH aggregation is frequent, raising up to 18% in subjects having a similarly affected first degree relative [24].

In addition, approximately 60% of the variation in serum alanine aminotransferase (ALT) as well as in circulating insulin concentrations, which are strictly correlated with hepatic fat content, are genetically determined in absence of other confounders, such as viral hepatitis or alcohol abuse, as yielded by the twin studies [25]. Loomba et al. demonstrated that both hepatic steatosis and fibrosis, non-invasively assessed, were tightly connected in monozygotic twins compared to dizygotic ones [26]. In a multivariate generalized model, adjusted for age, gender and ethnicity, the percentages of hereditability of hepatic steatosis and fibrosis were claimed at 52% and 50%, respectively. Moreover, in the same cohort, Cui et al. revealed a high degree (~75.6%) of shared genetic components between hepatic steatosis and fibrosis, irrespectively of environmental factors [27]. Likewise, cardiovascular comorbidities related to NAFLD, such as carotid plaques formation and abnormal intima-media thickness, have been reported to be strongly hereditable in a cohort of 208 adult Hungarian twins with NAFLD (63 monozygotic and 41 dizygotic pairs) [28].

The large disparity in NAFLD heritability which has been observed in different cohorts may be attributable to ethnicity [18,19]. Firstly, Wagenknecht and collaborators attested the much greater contribute (33%) of the genetic milieu on NAFLD onset in the Hispanic cohort belonging to the IRAS Family Study, compared to the African American one (14%) [22]. According to these findings, two large multi-ethnic population studies highlighted that Hispanics have a higher risk to develop NAFLD than Europeans [29,30]. Furthermore, there are discrepancies within the same ethnic group and amongst Hispanics, Mexicans have much higher prevalence of NAFLD compared to Dominicans or those from Puerto Rico [31]. Conversely, it has been confirmed the protection of African-Americans against NAFLD, irrespectively of T2D, overweight and socioeconomic factors, corroborating the role of heritability in NAFLD pathophysiology [11]. Indeed, African-Americans differed in the metabolic response to obesity and IR when compared to either Hispanics or Caucasians, resulting more resistant to triglyceride (TG) accumulation both in adipose tissue and in the liver [32].

A burgeoning number of heritable factors have been recognized as genetic modifier of NAFLD [11]. Specifically, Dongiovanni and colleagues, postulated that hepatic fat content constitutes the main driver of the evolution towards end-stage injuries in genetically predisposed subjects, thus indicating that each genetic variation exerts an effect on the spectrum of NAFLD, directly proportional to its ability to induce fat accumulation [33]. To date, the best known common inherited predictors of progressive NAFLD are the variants in *PNPLA3, TM6SF2*, *MBOAT7* and *GCKR* genes. However, given the challenging genetic framework of NAFLD, an impressive amount of novel inherited risk factors has been picked out through candidate gene association studies, genome wide association studies (GWAS) or exome wide association studies (EWAS). Thus, the most arduous challenge in the study of genetics of NAFLD is to postulate score-based systems which take into account polygenic determinants of NAFLD, that may guarantee the most highly predictive value, the best diagnostic accuracy and the more precise individualized therapy [34,35].

### 2.1. PNPLA3: Gambling on the Winning Horse

A turning point in our knowledge about the genetic contribution to NAFLD pathogenesis has been yielded by the first GWAS of NAFLD conducted in 2008 in a North American population of diverse ethnicity [36]. This screening allowed to identify, for the first time, the rs738409 C > G variant in *Patatin Like Phospholipase Domain Containing 3* (*PNPLA3*) gene, encoding the aminoacidic substitution isoleucine to methionine at the position 148 (p.I148M), as the genetic variant most tightly associated with hepatic fat accumulation. The risk effect of the rs738409 variant on fatty liver onset and progression towards more severe liver damage is the strongest ever reported for a common variant, attesting the proportion of the total variance attributed to this polymorphism at 5.3% [37]. Nowadays, the p.I148M PNPLA3 variation is still considered the most robust genetic predictor of the inter-individual and ethnicity-related differences in hepatic fat content and the primary risk factor for severe NAFLD.

The frequency distribution of the G minor allele is higher in Hispanics (49%) than in Europeans (23%) and less frequent in African Americans (17%), thereby justifying the higher prevalence of fatty liver in the former [36]. Consistently, the p.I148M variant confers a markedly increased odds to develop progressive NAFLD even in Asian populations. Even though the distribution of this variation is relatively high in Chinese individuals (around 30%), the prevalence of fatty liver is somewhat lower in East Asia, reaching the 25% in Japan, 18% in South Korea and 15% in China. However, due to lifestyle modifications, the incidence of NAFLD has been dramatically escalated in the last years. Thus, the control of weight gain and genetic assessment are strongly recommended even in both Asiatic adults and children [38].

*PNPLA3* gene codifies for a 481-aminoacid membrane lipase, located in the endoplasmic reticulum (ER) and at the lipid droplet (LD) surface in hepatocytes, adipocytes and in hepatic stellate cells (HSCs) [39,40]. An interaction between PNPLA3 and environmental factors exists. Indeed, its expression is modulated by sterol regulatory element-binding protein 1 (SREBP1c)/liver X receptor (LXR) and by carbohydrate response element binding protein (ChREBP), both activated by post-prandial or pathological hyperinsulinemia. Then, at post-transcriptional level, PNPLA3 protein levels are regulated by excessive amount of fatty acids, inhibiting their degradation [41,42]. Thus, sucrose and fructose over-consumption, physical inactivity and overweight foster the detrimental effect of the p.I148M variant [14,43,44]. As a consequence, the relationship between the rs738409 variant and NAFLD may be uncovered by the obesity [14]. Notwithstanding, the p.I148M variant boosts NAFLD towards NASH and fibrosis, even in lean subjects defined by BMI less than 25 kg/m^2^ [45].

In physiological conditions, PNPLA3 exerts its function hydrolyzing the lysophosphatidic acid to phosphatidic acid, whereas the p.I148M variant greatly troubles its enzymatic activity [46,47]. As a result, patients carrying the at-risk allele display elevated transaminases, accompanied by an enhanced incidence of all histological features related to fatty liver, including NASH, advanced fibrosis and HCC and modifying the response to therapeutic approaches [48]. Moreover, a high-throughput metabolic profiling of *PNPLA3* silenced Huh-7 cells demonstrated that *PNPLA3* depletion is associated with a global metabolic perturbation, reducing several amino acids and polyunsaturated fatty acids (PUFAs). Conversely, PNPLA3 p.I148M overexpression is associated with a 1.75-fold increase in lactic acid, suggesting a shift to anaerobic metabolism and mitochondrial respiratory chain dysfunction, supporting a critical role of PNPLA3 in the modulation of liver metabolism beyond its classical participation to TG remodeling [49]. Hence, hepatic specific overexpression of p.I148M in mice promotes steatosis and NASH, by priming the metabolic reprogramming and the activation of inflammatory pathways driven by either increased TG and ceramide species and reduced PUFA [50]. However, the role of PNPLA3 in lipid handling remains to be fully outlined.

Neither *Pnpla3* genetic defect nor *Pnpla3* wild-type (wt) over-expression in mice prompts steatosis [47,51,52], while mice carrying the p.I148M knock-in (KI) acquire fatty-laden hepatocytes upon a high-sucrose diet challenge [53]. In these mice, *Pnpla3* silencing improved the hallmarks of NAFLD and hepatic fibrosis [54]. In detail, it has been recently reported that the deleterious effect of the p.I148M variation is due to its interfering with the correct hydrolytic properties of other lipases, among which the adipose TG lipase (ATGL)/patatin-like phospholipase domain-containing 2 (PNPLA2) and directly by interacting with its cofactor, the comparative gene identification-58 (CGI-58) [55]. Indeed, p.I148M overexpression raised hepatic TG concentrations in wt, but not in Cgi-58 knock-out (KO) mice, indicating that the *PNPLA3* mutation may prompt hepatic steatosis, hindering ATGL/PNPLA2 activity on LDs in a CGI-58-dependent manner. Similar findings have been observed in brown adipocytes [56]. In addition, the p.I148M modification abolishes PNPLA3 ubiquitylation and proteasomal degradation resulting in the accumulation of the PNPLA3 mutated protein on LD surface, thus impairing TG mobilization and dampening TG dismissal [57,58]. BasuRay et al. engineered a PNPLA3 synthetic isoform that disentangles its two detrimental properties: the accumulation of the protein on LD and the loss of enzymatic activity [59]. In mice, the expression of an ubiquitylation-resistant form of Pnpla3 fosters the gathering of Pnpla3 mutated protein on hepatic LDs. In addition, in mice overexpressing Pnpla3 p.I148M the softening of Pnpla3 expression by shRNA or by or proteolysis-targeting chimera (PROTAC)-mediated degradation reduced TG storages [59]. In keeping with these findings, tissue expression of PNPLA3 is significantly enhanced in biopsies of patients carrying the p.I148M polymorphism, whereas the rare rs2294918 PNPLA3 (p.E434K) variant attenuates the impact of the p.I148M on steatosis and circulating liver enzymes in NAFLD patients, whereby down-modulating PNPLA3 expression on the LDs (up to 50%) [57,60]. In addition, Schwartz and collaborators demonstrated that momelotinib, a drug used in clinical trials to treat myelofibrosis, may represent an effective modulator of PNPLA3 expression, yielding >80% reduction in PNPLA3 mRNA levels and hampering intracellular lipid content in human primary hepatocytes and stellate cells [60]. Another possible mechanism underlying the TG engulfment in hepatocytes carrying the p.I148M variant is linked to the impairment of lipophagy in hepatocytes, thus dampening autophagic fluxes and LD degradation [61].

Luukkonen et al. [62], established, through a lipidomic approach, that TG in very-low density lipoproteins (VLDL) are depleted of PUFAs in p.I148M homozygous individuals during both fasting and feeding conditions. Therefore, in p.I148M hepatic cells, PUFA incorporation into TG is exacerbated and PUFA-containing diacylglycerols (DAGs) are gathered, at the expense of phosphatidylcholines (PCs) [62]. Hepatic lipid composition of DAG species may, in turn, greatly impact on insulin sensitivity. However, these modification in hepatic DAG composition has been not supported by Franko and colleagues [63], who corroborated the notion that *PNPLA3* variation is strictly correlated with fatty liver, but not with IR, whereby uncoupling these two NAFLD features. Indeed, IR-related NAFLD is marked by elevated concentrations of metabolically harmful saturated and mono-unsaturated TG, ceramides and free fatty acids (FFAs), whereas PNPLA3-related NAFLD by hepatic PUFA-containing TG. These observations may possibly clarify why metabolic NAFLD and not PNPLA3-related NAFLD, is tightly correlated with enhanced risk of T2D and cardiovascular comorbidities [64]. In addition, excessive deposition of PUFA-containing lipids has been noticed also in the adipose tissue of patients carrying the p.I148M, in whom the mutation did not modify the rate of lipolysis or the composition of circulating FFAs [65]. Conversely, recent findings indicate that PNPLA3 p.I148M confers an antiatherogenic plasma lipid profile in insulin-resistant individuals [66].

Intriguingly, PNPLA3 mutated protein may impair retinol release from HSCs [67], directly precipitating fibrogenesis and carcinogenesis irrespective of steatosis [68,69,70,71]. Indeed, NAFLD patients carrying the G allele harbor a peculiar histological pattern, depicted by exaggerated macro and microvesicular steatosis, portal inflammation, conspicuous proliferation of hepatic progenitor cells (HPCs), prominent ductular reaction, extensive myofibroblast and HSCs activation, thus sustaining portal fibers formation and severe systemic oxidative stress [72]. Furthermore, the different hepatic cell populations are characterized by various metabolic regulation of PNPLA3. Indeed, its expression is higher in α-SMA positive cells and it correlates with fibrosis stage in NASH patients [73].

Finally, the PNPLA3 p.I148M variant has been also reported to be associated with hepatic decompensation and liver-related death in a prospective study [74], with enhanced risk of fibrosis and HCC in patients affected by viral hepatitis or alcoholic liver disease (ALD) and with poor prognosis in patients affected by autoimmune hepatitis, regardless of steatosis [75,76,77].

### 2.2. TM6SF2 Loss-of-Function in NAFLD

In 2014, an EWAS ascertained the missense rs58542926 C > T variant in the *Transmembrane 6 superfamily member 2* (*TM6SF2)* gene which encodes the lysine to glutamate substitution at residue 167 (p.E167K) as a determinant of hepatic TG content, serum aminotransferases and lower serum lipoproteins [78]. *TM6SF2* gene codifies for a regulator of cholesterol biosynthesis, which acts in hepatic VLDL lipidation and assembly in the ER cisternae and in ER-Golgi compartments [78,79,80,81,82,83,84,85,86,87]. Smagris and colleagues highlighted for the first time in *Tm6sf2^−/−^* mice that TM6SF2 protein is essential for neutral lipid mobilization during VLDL construction [80]. Consequently, *TM6SF2* silencing alters hepatic lipid composition, affecting the synthesis of PC-containing PUFAs in both HuH7 cell lines and in human livers. The imbalance between PUFAs-conjugated PC and free PUFAs, in turn, impair VLDL gathering and induces the production of TG and cholesterol-esters clusters thus perturbing membrane dynamics [83,85]. O’Hare and colleagues explored the role of *TM6SF2* in small intestine of zebrafish and in Caco-2 enterocytes, and they observed that *TM6SF2*-deficiency induces LD buildup, decreases lipid clearance and favors ER stress [88]. Notably, it has been hypothesized that TM6SF2 may also play an enzymatic function converting zymosterol into 5-α-cholesta-7,24-dien-3β-ol, during cholesterol synthesis [82].

The TM6SF2 p.E167K substitution generated a misfolded protein which may run into rapid intracellular turnover and degradation, further determining its hepatic 50% downregulation in HuH7 hepatocytes [78]. *Tm6sf2* KO mice develop hepatic steatosis, caused by VLDL retention and they reduced circulating cholesterol [78]. Consistently, the presence of lower serum cholesterol and TG concentrations has been validated in individuals carrying the minor T allele in large cohort of NAFLD patients and in population-based studies such as the Dallas Heart Study (DHS), the Dallas Biobank and the Copenhagen Study [78,88,89]. In addition, Liu J Dajiang and collaborators found more than 400 coding and noncoding variants which influenced metabolic traits and plasma lipids. Among them, the p.E167K resulted as one of the causal variants that mainly mitigated circulating TG levels and increased risk of fatty liver and T2D [90].

Several studies indicated that the p.E167K mutation strikingly impacts on hepatic TG content, causing higher degree of steatosis, and also it was associated with elevated transaminases and with histological inflammation, ballooning and fibrosis in both pediatric and adult patients [91,92,93,94]. Interestingly, the missense rs58542926 polymorphism conferred protection against cardiovascular complications, hampering serum low density lipoprotein (LDL) concentration. Overall, these findings have supported the hypothesis that the minor T allele may defend against cardiovascular events, but it worsens liver disease severity in NAFLD patients [89,95,96].

The impact and the prevalence of the p.E167K mutation differs across the ethnic groups and across subjects characterized by different visceral adiposity. In particular, it has been reported that it modifies the risk of fatty liver only in Caucasian and Afro-American obese children [92]. Furthermore, in lean biopsied NAFLD patients the p.E167K allele predisposes to IR in both hepatic and adipose tissues [97]. Notably, the *TM6SF2* rs58542926 variant has a higher prevalence in non-obese normo-lipemic patients compared to obese ones [98].

The majority of data supported that *TM6SF2* loss-of-function alters lipid metabolism and increases the susceptibility to NAFLD spectrum [89,94,99], although its association fibrosis and HCC is still controversial [86,99,100]. For example, Sookoian and coworkers did not find any associations between the *TM6SF2* mutation and transaminases, inflammation and fibrosis in 361 NAFLD individuals possibly due to the low frequency of the polymorphism and lack of statistical power [100]. Conversely, Liu et al. reported that the T risk allele conferred an increased predisposition to develop NAFLD-related advanced fibrosis in two independent cohort regardless of other confounders as gender, sex, BMI, T2D and *PNPLA3* rs738409 genotype [101]. The association between the rs58542926 variation, hepatic fibrosis and HCC was further observed in a cross-sectional and in a small cohort studies including 502 and 129 NAFLD patients, respectively [99,102]. Finally, a meta-analysis considering 24,147 individuals with heterogeneous chronic liver disorders, associated the p.E167K polymorphism with higher risk of cirrhosis and HCC rather than viral hepatitis, especially in alcohol abusers [103,104]. In keeping with this evidence, the p.E167K variant correlated with alcohol-related HCC in 511 cirrhotic patients and in a prospective cohort of 249 ALD patients [105,106].

### 2.3. MBOAT7: A Common Modifier of Liver Damage

MBOAT7, also known as lyso-phosphatidylinositol (Lyso-PI) acyl-transferase1 (LPIAT1), is an enzyme that participate to the “Lands’ Cycle” of phospholipid acyl-chain remodeling of the membranes. MBOAT7 is highly expressed in human hepatocytes, sinusoidal endothelial cells, immune cell subsets, HSCs and less expressed in cholangiocytes [107,108,109]. It is localized in the membrane bridging ER and mitochondria in which LD and fat biosynthesis occur. It conjugates an acyl-CoA to the second acyl-chain of lyso-phospholipids, using arachidonoyl-CoA as substrate. Therefore, it regulates phospholipid desaturation and free arachidonic acid levels, precursor of dangerous eicosanoids [110]. As a matter of fact, in neutrophils MBOAT7 activation has been related to anti-inflammatory processes, by limiting the availability of free arachidonic acid for the synthesis of Leukotriene B4, a strong chemoattractant mediator [111].

MBOAT7 is involved in multiple aspects of neuronal development in brain, where arachidonic acid is the most enriched PUFA. Mboat7 KO mice die within a month and show a severe neuronal impairment [112,113]. Consistently, inactivating variants in *MBOAT7* lead to intellectual disability accompanied by epilepsy and autistic features in patients [114,115].

In 2015, the first GWAS regarding the inherited determinants of cirrhosis in heavy drinkers, identified the common rs641738 C > T variant close to MBOAT7, as a novel mediator of the susceptibility to hepatic injuries [116,117]. Afterwards, Mancina and Dongiovanni, corroborate these findings, demonstrating that the rs641738 variant associates with the predisposition towards hepatic fat accumulation and to the entire phenotypic umbrella of liver injuries related to NAFLD, among which HCC [107,118,119].

*MBOAT7* variant was linked to elevated total body fat percentage, transaminases and C-reactive protein levels in pediatric individuals [120] and with enhanced ALT and more severe steatosis and fibrosis in children with NAFLD, showing a synergistic effect with PNPLA3 p.I148M, and TM6SF2 p.E167K variants on pediatric NAFLD risk [121], also confirmed in adult patients [34,35,122].

Noteworthy, the rs641738 inherited variation has also been as associated with early fibrosis in viral hepatitis B and C, constituting a shared modulator of liver injuries [123,124]. Even more, rare loss-of-function variations in *MBOAT7* have been found to be associated with HCC in NAFLD patients [125]. The association between the rs641738 variant and liver abnormalities remains disputed, mainly due to the different sample size and ethnicity of the cohorts enrolled in the studies or to the diverse assessment of hepatic steatosis [126,127,128,129,130]. However, a meta-analysis validated the associations between the rs641738 variant and liver fat, ALT, histological severity of NAFLD, fibrosis and HCC at least in individuals of European descent [131]. Specifically, it has been reported that in T allele carriers, the total risk of NAFLD, advanced fibrosis and HCC is attested at 20%, 30% and 40% more compared to non-carriers, respectively.

Mancina and Dongiovanni have widely showed that the mechanisms underlying these associations is related to blunted hepatic MBOAT7 gene and protein expressions, thus perturbing PI species composition [109,118], as then supported by Luukkonen’s observations [119]. According to the impaired hepatic MBOAT7 enzymatic activity, patients carrying the T allele displayed changes in plasma and hepatic PI species, decreasing specifically PI enriched in omega-3 PUFA and arachidonic acid [118,119]. However, these remarks have been not fully replicated by Sookoian, which demonstrated that MBOAT7 is down-regulated in NAFLD even independently of the rs641738 polymorphism [127].

This notion was strongly reinforced by our recent manuscript, that pinpoints that hepatic MBOAT7 down-regulation is a maladaptive response to hyperinsulinemia and that its hampered enzymatic activity forces hepatic fat storage in patients, in *in vivo* models representative of NAFLD and in MBOAT7 silenced HepG2 hepatoma cells (also referred to as *MBOAT7^−/−^*) [109,132]. Specifically, in presence of severe obesity and hyperinsulinemia, MBOAT7 is downmodulated both in patients and in rodents, independently of the genetic background. A possible link between MBOAT7 and IR has been provided even by Helsley and colleagues [133], who confirmed MBOAT7 suppression during IR and by Umano and coworkers, who correlated lower degree of whole-body insulin sensitivity in obese children and MBOAT7 [134].

In mice, acute MBOAT7 silencing conveys hepatic fat entrapment and *MBOAT7^−/−^* hepatocytes acquire a cell-autonomous property to accumulate giant LDs, supporting the idea that MBOAT7 may be causally involved in steatosis onset. Indeed, a derangement in MBOAT7 function contributes to accumulate saturated phospholipids, mainly, PI species that may be shunted to saturated and mono-unsaturated TG synthesis, further sustaining fatty-laden hepatocyte formation [109,132]. In line with this data, *MBOAT7* breakage hustles the induction of lipogenic program, due to ER stress and to the activation of SREBP-1c, a transcription factor that coordinates the activation of genes involved in fatty acid biosynthesis [135]. The causative role of MBOAT7 in fatty liver has been independently reported by Helsley [133] and then by Tanaka [136].

Notwithstanding, MBOAT7 depletion in 3D-spheroids composed by hepatocytes and HSCs, stimulated cytokines secretion, fibrogenic markers and collagen deposition [136], due to the accumulation of the MBOAT7 substrate Lyso-PI lipids [133]. Indeed, circulating saturated Lyso-PI were found to be substantially elevated in patients affected by severe fibrosis compared to healthy individuals. In turn, Lyso-PI administration may promote hepatic lobular inflammation and fibrosis in *MBOAT7* deficient mice, but not in their wt littermates [133]. Notably, this data has been further strengthened by Fondevila et al., who revealed that the increased serum Lyso-PI levels in obese NASH patients fuel the hepatic over-expression of the G protein-coupled receptor 55 (GPR55), a putative cannabinoid receptor [137]. Moreover, Lyso-PI treatment in mice and in cultured cells activated lipogenic genes and HSCs trans-differentiation, in a GPR55-dependent fashion. GPR55 deficiency ameliorated hepatic injuries in mice fed high fat, methionine low, choline deficient diet or injected with carbon tetrachloride (CCl_4_). Disturbances of the PI side chain reshaping in hepatocytes alone is sufficient to elicit spontaneous steatosis, and fibrosis upon a dietary induction [138].

Taken together, these observations point out that the restoration of MBOAT7 activity or a reduction of its effectors may constitute possible therapeutic interventions to manage NAFLD patients [139,140,141].

### 2.4. GCKR: The Jointing of Glucose Handling and Fatty Liver

Together with the already discussed *PNPLA3, TM6SF2* and *MBOAT7* variants, another common loss-of-function inborn mutation has been afterward identified. Specifically, the rs1260326 C > T variant in the *GCKR* gene, encoding the P446L aminoacidic substitution has been broadly coupled with heightened fasting TG, enlarged VLDL particles, fatty liver and metabolic abnormalities [21,130,142,143]. *GCKR* gene codifies the glucokinase regulatory protein, which is implicated in the regulation of glucose homeostasis and glycemic control, whereby modulating glucose influx into the hepatocytes and the consequent induction of *de novo* lipogenesis (DNL). For this reason, alterations of *GCKR* impair glucokinase redistribution between the cytosol and nucleus, thus tackling its negative modulation in response to fructose-6-phosphate and in turn, they constitutively induces glucose uptake into the hepatocytes [144]. Unrestricted hepatic glycolysis associated with carriage of the minor 446L allele leads on one hand to lower glucose and insulin levels, but on the other hand to extended malonyl-CoA concentrations, which in turn may favor hepatic fat accumulation by serving as a substrate for lipogenesis and by blocking fatty acid oxidation through the inhibition of carnitine-palmitoyl transferase-1. In details, it has been shown that overweight adolescents carrying the *GCKR* rs1260326 in homozygosity exhibit enhanced lipid assembly, as a consequence of exasperated glycolytic carbon flux to TG synthesis and the effect of the minor T risk allele was impressively amplified by adiposity [14,145].

The co-presence of the two common *PNPLA3* and *GCKR* at-risk alleles may favor the gathering of TG produced by the conversion of carbohydrate in more packed LDs. These alterations may justify up to 32% of variability in hepatic fat deposition in Caucasian obese children, 39% in African-Americans and 15% in Hispanics [143]. The additive effect of the *GCKR* and *PNPLA3* variants escalated the NAFLD [146], NASH and HCC odds [146,147]. Moreover, the *GCKR* variant increased the susceptibility to fibrosis onset coupled with increased circulating TG in adult NAFLD patients, without affecting LDL and HDL cholesterol levels and the risk of coronary artery disease (CAD) [142,148].

### 2.5. Protective Inheritable Determinants: The HSD17B13 and PPP1R3B Variations

In 2018, the splice variant (rs72613567:TA) in *HSD17B13* gene, encoding the hydroxysteroid 17-β dehydrogenase 13, was discovered to be associated with protection against histological steatohepatitis, fibrosis and cirrhosis in both NAFLD and ALD patients [149]. The rs72613567 is an insertion of an adenine close to the donor splice site of the last exon (TA allele), hesitating in a truncated transcript, diminished mRNA and protein levels and strongly compromised enzymatic activity of HSD17B13, which is localized on the LD surface into the hepatocytes alongside PNPLA3 [149,150,151]. The biological role of HSD17B13 remains yet-to-be-understood. However, it has been demonstrated that HSD17B13 is over-expressed on the LDs, in NAFLD subjects and in preclinical NASH models [151]. Indeed, HSD17B13 induction exacerbates the amount and size of LDs in hepatocytes [151]. On the contrary, *HSD17B13* knock-down mice develop steatosis directly prompting lipogenic program in a SREBP-1c and fatty acid synthase (FAS)-dependent fashion [151]. As so far, the impact of HSD17B13 on hepatic fat content is still under definition and the protective rs72613567 variation has never been associated with an amelioration in TG content [149]. Furthermore, Kozlitina et al. [152], identified another loss-of-function inherited modification in the *HSD17B13* gene (c.573delC, rs143404524), more common in African-Americans than in Hispanics or Caucasians. The latter might be responsible for protection against the development of chronic liver injuries. In a case-control study, Pirola et al. [153], stated that TA allele offers protection against histological NASH and fibrosis in NAFLD patients. This notion has been further corroborated in a recent GWAS which described a stronger protective effect of the *HSD17B13* variant in the context of steatohepatitis rather than fibrosis [130].

The likely mechanism which underlies these genetic associations seems to be due to an increased concentrations of hepatic phospholipids in carriers compared to non-carriers, that is coupled to a reduction of pro-inflammatory genes [154]. Furthermore, the rs72613567 loss-of-function mutation has been correlated to decreased transaminase levels and to a reduced risk of HCC in 111,612 subjects belonging to the Danish general population and in 3315 European descent, respectively [155,156]. According to these findings, among differentially expressed genes related to HCC glycolysis, *HSD17B13* has been emerged as critical modulator of this metabolic process. Enhanced cellular glycolysis for energy production predicts adverse clinical outcomes and poor prognosis in many types of human cancers, especially HCC. Indeed, cancer cells primarily exploit this via in response to exaggerated energy demand to support cell survival and rapid proliferation (phenomenon known as Warburg effect). Therefore, modulation of HSD17B13 expression and activity might potentially represent a new avenue to design targeted therapies for the treatment of HCC [157].

An intriguing interaction between *HSD17B13* rs72613567 and PNPLA3 p.I148M has been described. Indeed, the *HSD17B13* TA allele mitigates the impact of the p.I148M variant on liver damage, although it does not ameliorate hepatic fat accumulation [149]. Moreover, the lowering effect of *HSD17B13* variant on transaminases is amplified in carriers of the p.I148M allele [155]. A very recent high-throughput screening of circulating metabolites revealed that risk alleles in *PNPLA3* and in *HSD17B13* were both associated with higher 3-methylglutarylcarnitine and reduced levels of Lyso-PCs [158]. In sum, this data suggests that HSD17B13 modulation especially in patients carrying the *PNPLA3* G allele may constitute a potential therapeutic approach in the management of chronic liver diseases.

An alternative protective player against hepatic disorders is exemplified by the rs4841132 G > A variation, which strengthens the expression of the Protein Phosphatase 1 Regulatory Subunit 3B (*PPP1R3B)* gene, involved in glycogenesis [21,159]. As a consequence, although it is responsible for a reduced NAFLD risk, it may facilitate glycogen synthesis and storage [159,160]. Indeed, in preclinical models, hepatic genetic deficiency of *PPP1R3B* lowers the abundance of glycogen synthase, glucose incorporation into glycogen, total hepatic glycogen levels and fasting plasma glucose [161]. The complex impact of *PPP1R3B* variation on steatosis and progressive liver injuries is still debated. Notwithstanding, Dongiovanni and colleagues [160], elucidated that the rs4841132 variant is associated with protection against steatosis and fibrosis, hesitating into a reduced risk of HCC in patients with NAFLD, but not in individuals from general population.

## 3. Genetic Signature of Glucose and Lipid Metabolism in NAFLD

In the last decades, it clearly emerged that IR is a key player in NAFLD pathogenesis [162,163]. In particular, IR strongly predicts the severity of hepatic fibrosis [162], the main determinant of NAFLD prognosis [164], and advanced fibrosis often occurs in NAFLD patients with T2D, even independently of inflammation and NASH [165,166].Therefore, genetic variants that suppress the activation of insulin signaling may induce fibrosis in NAFLD [167]. The rs1801278 (G972R) loss-of-function mutation in insulin receptor substrate (*IRS1*) and the gain-of-function one in the ectonucleotide pyrophosphatase/phosphodiesterase1 (*ENPP1*) 121Q genes were both related to dyslipidemia, obesity and hepatic fibrosis [167]. On the contrary, the rs2954021 variant in tribbles homolog1 (*TRIB1*), involved in the modulation of hepatic glycogen storage, affected plasma glucose, TG and cholesterol levels [168].

Similarly, other variations in genes governing hepatic lipid handling and release predispose to fatty liver. For instance, variants within Apolipoprotein B (*APOB*), involved in VLDL organization and secretion, have been associated with a protection against cardiovascular complications, due to the lowering of circulating lipoproteins and in turn, they favor severe hepatic fat depot formation, that may foster the progression of liver injury up to HCC [125,169]. Moreover, even microsomal triglyceride transfer protein (*MTTP*) inherited alterations may prompt VLDL retention [170].

In addition, two common promoter variants in the apolipoprotein C3 (APOC3) (*APOC3* T-455C and C-482T), a component of chylomicrons, VLDL and HDL cholesterol particles, may predispose to steatosis in Indians, but not in other ethnic groups, supporting the notion that genetic factors modulating TG metabolism outside of the liver are less implicated in the onset of progressive NAFLD [171,172,173].

Likewise, Dongiovanni et al. [174], elucidated that the proprotein convertase subtilisin/kexin type 7 (*PCSK7*) rs236918 G > C variant affects fasting lipids and hepatic injury in a large cohort of NAFLD subjects, coupling atherogenic dyslipidemia with NASH and hepatic fibrosis. By stratifying patients according to the presence of the PNPLA3 p.I148M allele, the *PCSK7* rs236918 polymorphism was associated with advanced steatosis. Indeed, in hepatoma cells carrying the p.I148M allele in homozygosity, *PCSK7* genetic deficiency decreased the expression of genes involved in DNL, inflammation and fibrosis, even after FFA supplementation [174]. Furthermore, Huang et al. [175], revealed a correlation between the *PCSK7* at-risk allele, hyperinsulinemia and homeostatic model assessment for IR (HOMA-IR) after a high-carbohydrate challenge. *PCSK7* is strongly implicated in lipid homeostasis, since it is localized in a genomic region close to the gene cluster *APOA5/APOA4/APOC3/APOA1*, involved in lipoprotein metabolism regulation. In keeping with these findings, *Pcsk7^−/−^* mice fed HFD are characterized by elevated plasma apolipoprotein concentrations and enhanced lipoprotein lipase (Lpl) adipose tissue activity [176].

Even more, aberrancies in another member of the proprotein convertase subtilisin/kexin family, PCSK9, have been widely associated with hereditary hypercholesterolemia [177], severe fat deposition [178] and cardiovascular abnormalities [179], due to its impact on LDL uptake. PCSK9 is a nutrient sensor, and it is strongly influenced by nutritional *status*. Indeed, its expression declines in mice after 24 h of fasting. On the contrary, PCSK9 mRNA levels are renewed through SREBP-1c and DNL activation upon high carbohydrate refeeding or insulin stimulation [180]. Loss-of-function mutations in *PCSK9* diminish plasma LDL cholesterol, without inducing steatosis [181]. For example, the *PCSK9* rs11591147 (p.R46L) loss-of-function variant blunted LDL levels and protected against NAFLD, NASH and fibrosis, irrespectively of confounders [182]. Conversely, gain-of-function alterations of *PCSK9* as the rs7552841 variant lead to familial hypercholesterolemia and enhance CAD risk.

Hepatic dysfunctions may be caused even by rare mutations of lysosomal acid lipase (*LIPA*) gene, that induce lysosomal acid lipase (LAL) defects. LAL participates to the hydrolysis of cholesteryl esters, TG and LDL into free cholesterol and fatty acids. Its functional aberrancy fosters un-hydrolyzed compounds accumulation into the hepatocytes, whereby favoring atherogenic dyslipidemia, hepatic steatosis and severe fibrosis [183,184]. In turn, LAL restoration using recombinant sebelipase alpha administration in patients for up to 5 years may improves liver enzymes, hepatic features of NAFLD and circulating lipids in patients (clinical trial NCT01488097) [185,186].

Finally, even variants that alter FFA fluxes into the liver or their catabolism, such as the rs56225452 in fatty acid transport proteins (*FATP5*) or the rs13412852 in Lipin1 (*LPIN1*), may leverage IR and steatosis [187,188].

## 4. Genetics of Lipid Droplets

LD accumulation in the liver is the primary hallmark of NAFLD. Compelling evidence indicates that LDs should not be considered as just ‘innocent bystander’, but conversely, they participate to multiple processes that lead to NASH. LDs are enormously dynamic, modifying their localization, size, lipid and protein composition in response to environmental *stimuli* and energy demand. Hence, they are engaged not only in energy expenditure to produce ATP but also in signaling pathways, acting as hubs that integrate metabolic and inflammatory processes.

Genetic risk factors may play a crucial role as modifiers of lipid composition and LD dimensions, whereby causing the alteration of the expression of LD-associated proteins, which regulates lipid storage. Thus, together with the above-mentioned p.I148M PNPLA3 and *HSD17B13* rs72613567 variants, many other polymorphisms in genes implicated in LD handling have been recently associated with NAFLD. Among them, Perilipin-2 (PLIN2) rs35568725 (Ser251Pro) variant has been associated with IR and atherosclerosis, in two population studies. In particular, PLIN2 protein participates to the formation, stability and trafficking of LDs and in VLDL lipidation. The Ser251Pro mutation induces smaller, but more numerous LDs in hepatocytes, resembling microvesicular steatosis and conveying the risk of NASH in NAFLD patients [189]. The increasing number of small LDs was not translated into enhanced cellular capacity to store fat, but more so into high number of LD-associated proteins on the surface area. Thus, we could speculate that the presence of this variant may enhanced the risk related to PNPLA3 p.I148M carriage in patients. Even more, *PLIN2* variant has been associated with reduced circulating TG and VLDL [190].

Similarly, the common noncoding polymorphism, rs884164 in another LD-associated gene, *PLIN5,* causes a down-regulation of PLIN5 expression, a protein that facilitates the association between LDs and mitochondria. The recruitment of mitochondria to LDs during conditions of high substrate availability may favor lipid catabolism. Thus, PLIN5 hampered expression is associated with a poorer outcome following myocardial ischemia and *PLIN5* deficiency is related to increased oxidative stress in cardiomyocytes [191,192].

Since the degradation of cellular lipids is mediated by a selective autophagic process, named lipophagy, an impairment in this mechanism induced by genetic defects may dampen lipid β-oxidations, accelerating their accumulation. For instance, the rs10065172 variant in the autophagy-related *IRGM* gene may increase the risk of developing steatosis and *IRGM* knockdown inhibits autophagic flux and increases LD content in HepG2 cells [193]. IRGM is generally localized on endosomes/lysosomes, while in HFD-fed mice it co-localizes with ATGL/PNPLA2 at LD surface, where it recruits autophagic mediators, such as LC3B, in attempt to counteract to steatosis development. Hence, IRGM overexpression protects against hepatic lipid storage [194].

## 5. Advanced Liver Injuries and Genetic Variants

A series of stressful triggers may precipitate fatty liver up to NASH and severe fibrosis. Among them are included oxidative stress fostered by reactive oxygen species (ROS) overproduction, intracellular organelle derangement, i.e., ER and mitochondrial abnormalities and dysfunctions, innate immune inflammation and pro-inflammatory cytokine and chemokine release [195], intestinal high permeability and gut-derived harmful by-products (due to leaky gut) [196], and HSCs activation to myofibroblasts [197].

Interleukin 28 (*IL28*) gene codifies for the interferon λ3/λ4 (IFNL3/4), and the rs12979860 CC variant has been associated with interferon λ3 over-production [198]. The latter mediates the clearance of hepatitis C virus, and more aggressive NASH and fibrosis in NAFLD patients [199,200]. In particular, it has been yielded a genetic model to predict significant fibrosis, named FibroGENE, that includes the rs12979860 variant, age, gender and the routinely assessed clinical and biochemical parameters [201]. The rs12979860 is in linkage disequilibrium with the another variant, the *IFNL4* rs368234815 TT > δG. Patients carrying the rs368234815 TT allele are predisposed to develop higher degree of lobular inflammation and fibrosis compared to non-carriers [202]. Contrasting findings have been observed in carriers of the rs3480 A > G variant in the fibronectin type III domain-containing protein 5 (*FNDC5*) gene, encoding irisin, a myokine, that intervenes in HSCs activation and collagen deposition [203,204]. The minor G allele predisposes to elevated extents of steatosis, likely by modulating irisin expression [204]. Likewise, the rs2228603 polymorphism in Neurocan, the rs12137855 variation in lysophospholipase-like 1 (*LYPLAL1*) and the rs10883437 SNP close to the carboxypeptidase n subunit 1 (*CPN1*) have been coupled to severe NAFLD [205,206].

Alongside, mounting evidence indicates that the gut-derived fibroblast growth factor (FGF) 19, engaged in lipid and carbohydrate metabolism in response to nutritional *status* through the binding to its hepatic receptor, fibroblast growth factor receptor 4 (FGFR4), is involved in metabolic diseases and NAFLD [207]. Dongiovanni and Crudele et al. [208], demonstrated that the rs17618244 G > A variant in the β-Klotho (*KLB*) gene, encoding the hepatic co-receptor of FGFR4, dampened KLB plasma levels, leading to inflammation, ballooning, fibrosis and to the over-expression of genes involved in lipotoxicity in overweight NAFLD pediatric patients [208]. Furthermore, KLB complexing with others FGFRs also binds the hormone FGF21, released from the liver and adipose tissue. In detail, FGF21 is implicated in glucose and TG uptake by white and brown adipose tissue, through the interaction with FGFR1 [209]. However, FGF21 circulating levels are paradoxically increased in obese patients and in those with NAFLD, as a protective response to KLB down-regulation and to NAFLD-induced adverse effects, e.g., lipotoxicity, oxidative and ER stress [210,211]. Thus, KLB/FGF19/FGF21 pathway may represent a druggable target in NAFLD patients through the rescue of KLB levels.

Concerning the development of fibrosis, the alternative splicing of the Krueppel-like factor 6 (*KLF6*) gene, that is expressed by the HSCs during their transdifferentiation, associates with mild NAFLD and reduced fibrosis [212]. Conversely, variants in *HFE* and *TMPRSS6* genes likely by predisposing to hepatic iron depot formation are correlated with more severe fibrosis in NAFLD patients [213].

The rs4374383 non-coding variant in the macrophage c-mer tyrosine kinase (*MERTK*), a tyrosine kinase that initiates the removal of dying cells by phagocytes and that mediates HSCs activation, protects against fibrosis in both NAFLD and in viral hepatitis C, eliciting MERTK down-modulation [214,215]. Consistently, it has been stated that MerTK cleavage in hepatic macrophages is reduced during the transition from simple steatosis to NASH, promoting transforming growth factor β (TGF-β) release and HSCs activation [216]. Novel insights into the role of MERTK in metabolic processes, has been brilliantly proposed by Nicolás-Ávila and colleagues [217], which demonstrated that macrophages may actively entrap materials, including dysfunctional mitochondria ejected from injured cardiomyocytes through dedicated membranous particles enriched in phosphatidyl-serine (PS), with the purpose to maintain the global tissue homeostasis. This peculiar process occurs in MERTK-dependent manner, and it is driven by the cardiomyocytes’ autophagy machinery, prompted by cardiac stress. Thus, MERTK depletion abolished the removal of the exhausted mitochondria, hindered autophagic processes and resulted in the inflammasome and autophagy arrest, ultimately compromising mitochondrial fitness. Thus, this novel non-canonical route for the extrusion of cellular waste, including abnormal mitochondria and other organelles into the extracellular space, then scavenged by resident macrophages, may pave the way to potential translational implications on the study of other tissues characterized by high mitochondrial biomass and energy demand, in both healthy and disease status.

Finally, the susceptibility to fibrogenesis and carcinogenesis is also influenced by cellular senescence and cell cycle arrest. Therefore, the rs762623 in cyclin dependent kinase inhibitor 1A (*CDKI1A*) which encodes the cellular senescence marker p21, was significantly associated with disease progression in NAFLD [218]. Likewise, telomerase reverse transcriptase (*TERT*) gene loss-of-function mutations associated with familial cirrhosis and accelerated HCC [219]. Similarly, the rs599839 A > G variant, which causes the overexpression of the oncogene Proline And Serine Rich Coiled-Coil 1 (*PSRC1*), has been associated with enhanced HCC risk in NAFLD patients, irrespectively of fibrosis severity, and with poor prognosis and advanced tumor stage [220]. Even more, the Neurotensin (*NTS*) rs1800832 variant predisposes to cirrhosis and HCC in NAFLD patients likely by affecting NTS protein activity [221].

## 6. Mitochondrial Dysfunctions: The Tipping Point in the Switching from Simple Steatosis to Steatohepatitis

Growing evidence pinpoints the critical role of organelle abnormalities in the switching from fatty liver towards NASH. Mitochondrial anomalies are closely entangled into the pathogenesis of NAFLD so much so that it has been considered as a mitochondrial disease [222]. During early stages of NAFLD, mitochondrial activity and biomass is adapted in response to IR and to fat accumulation. However, sustained mitochondrial oxidative flux hesitates in exasperated ROS production, triggering phospholipid lipoperoxidation, cellular stress and mitochondrial DNA damage, tissue inflammation and cell death which may precipitate the progression to NASH and more advanced liver injuries [223].

In this context, the knowledge of genetically determined mitochondrial dysregulations may be determinant to predict the course of the disease. Indeed, common polymorphisms in genes regulating mitochondrial homeostasis have been associated with NAFLD and to its progressive forms. For instance, the rs4880 C47T variant in the superoxide dismutase 2 (*SOD2*) gene, encoding the antioxidant enzyme manganese superoxide dismutase, results in a Valine to Alanine substitution in the signal region addressing the protein to the mitochondrial matrix, where it exerts its function, and the T allele has been related to increased enzymatic activity. Thus, an higher frequency of *SOD2* T/T genotype in biopsy-proven NASH patients compared to healthy controls has been reported [224]. This variant has been further associated with severe fibrosis in NAFLD patients, as a proof of concept that mitochondria-derived oxidative stress is required for fibrosing NASH onset [225].

Alongside, the homozygosity for the −866 G > A mutation in the promoter region of the uncoupling protein 2 (*UCP2*) gene protects against NASH, whereby enhancing hepatic UCP2 expression [226]. The latter is implicated in the regulation of mitochondrial lipid efflux and oxidative metabolism and its hepatic expression increased in NASH patients causing a proton leak and a reduction of redox pressure on the mitochondrial respiratory chain, protecting the hepatic tissue against liver damage worsening [227].

Conversely, a non-coding variant in the promoter (−55C > T, rs1800849) of another member of the UCP family, the uncoupling protein 3 (*UCP3*) gene has been correlated with low insulin sensitivity, IR, reduced adiponectin secretion, moderate-severe hepatic steatosis and inflammation in obese NAFLD individuals [228]. UCP3 is a mitochondrial proton transporter that protects against fatty acid-mediated oxidative stress, uncoupling the oxidative phosphorylation by increasing the proton leak of the inner mitochondrial membrane.

Sirtuins (SIRTs) are a family of nicotinamide adenine dinucleotide (NAD^+^)-dependent deacetylases embroiled in cellular metabolism. There are 7 distinct SIRTs in mammals (SIRT1–7), which share the catalytic core domain, but they have different subcellular localizations. Indeed, SIRT1, SIRT6 and SIRT7 are mainly localized into the nuclei, SIRT2 is primarily found into the cytoplasm, while SIRT3, SIRT4 and SIRT5 have a mitochondrial distribution [229]. SIRTs along with UCPs may modulate oxidative stress thereby influencing the risk of subclinical atherosclerosis and cardiovascular complications. Indeed, it has been demonstrated that the *SIRT6* rs107251 and the *SIRT5* rs12216101 were associated with an elevated susceptibility to carotid plaques formation, whereas carriers of the T allele of *UCP5* rs5977238 had a lower risk, in 1018 stroke-free subjects from the Northern Manhattan Study (NOMAS) [230]. Even though cardiovascular abnormalities are recurrent in NAFLD patients, the implication of SIRTs genetic variations in this context remains to be fully elucidated.

**Figure 1:**
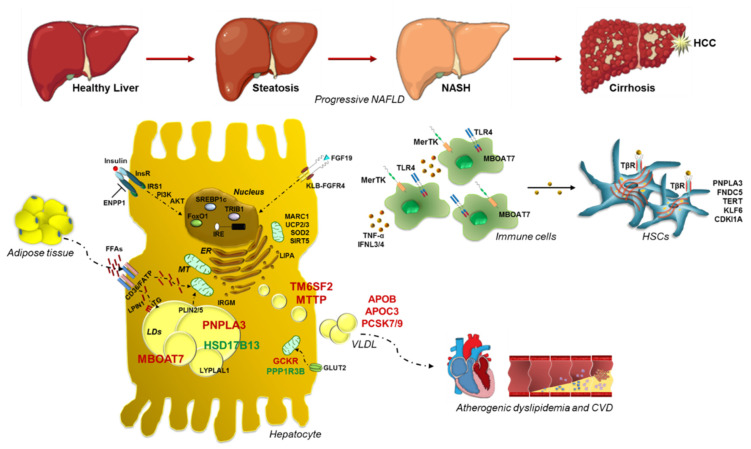
Impact of genetics in NAFLD pathogenesis and progression towards advanced liver damage. Schematic illustration of the most relevant inherited variations involved in progressive NAFLD, shedding light into their functional effects. PNPLA3, localized at the LD surface in hepatocytes, catalyzes TG hydrolysis. The p.148M variant enhances hepatic TG content upon mutant protein accumulation, hampering TG turnover and dismissal. TM6SF2 is implicated in VLDL formation in ER and release, whereas MBOAT7 transfers arachidonoyl-CoA to Lyso-PI, maintaining membrane fluidity. Their variations dampen VLDL secretion and membrane dynamism, respectively. *Viceversa*, genetic variants in *HSD17B13* and *PPP1R3B* may exert a protective effect against NAFLD. Heritable variations may also influence glucose and insulin signaling, FFA uptake, fat deposition and VLDL turnover, precipitating fatty liver. In addition, IR and elevated FFAs derived from adipose tissue lipolysis exacerbate fat depot formation induced by genetic modifiers, even activating DNL. Recently, common SNPs in modulators of mitochondrial (MT) function have been proposed as active players in the switching from steatosis to NASH and fibrosis, further corroborating the role of organelle abnormalities in these processes. Furthermore, variants in genes regulating inflammatory response and HSCs activation may precipitate fatty liver to worsened conditions. Finally, genetically determined perturbations in circulating lipids may trigger cardiovascular comorbidities. Dotted lines refer to influx and efflux processes into the hepatocyte, whereas solid lines refer to cell activation or to the transition from simple steatosis up to cirrhosis-HCC.

More recently, a novel common missense variant (rs2642438 A165T) in the mitochondrial amidoxime-reducing component 1 *(MARC1)* gene has been identified. *MARC1,* also known as *MTARC1* or *MOSC1,* encodes the mitochondrial amidoxime reducing component 1, a molybdenum-containing enzyme that regulates endogenous nitric oxide levels and biosynthesis, catalyzing the conversion of nitrite to produce nitric oxide. The A165T variant is located at the N-terminal domain which anchors the protein to the outer membrane of the mitochondria. The threonine to alanine aminoacidic substitution results in a truncating protein making the rs2642438 a loss-of-function mutation. The A165T variant has been associated with protection against all-cause cirrhosis, reduced hepatic fat content and lower levels of liver enzymes [231]. Specifically, in patients affected by alcohol-related cirrhosis *MARC1* and heterogeneous nuclear ribonucleoprotein U like 1 gene (*HNRNPUL1)* variations has been emerged as risk modifiers of liver damage, in a GWAS of samples from the United Kingdom Biobank [232]. Afterwards, Luukkonen and collaborators [233] investigated the effect of the rs2642438 variant on the severity of NAFLD and they demonstrated that patients carrying the A165T allele had markedly lower prevalence of inflammation and fibrosis, compared to non-carriers. This effect seems to be due to the precise lipid signature that describes A165T allele carriers, displaying increased levels of hepatic polyunsaturated-PC similarly to carriers of the *HSD17B13* rs72613567 variant and opposite to what the same authors observed in *PNPLA3* p.I148M carriers. According to these observations, the wt forms of *MARC1* are related to higher levels of sphingomyelins (i.e., C20:2), Lyso-PC (C14:0 and C15:0) and PC (C34:1 and C40:2) compared to patients carrying the A165T allele, thereby confirming the presence of a distinctive metabolomic pattern by using comprehensive metabolomics data from two population-based studies, including 9135 participants from the Fenland study and 9902 participants from the EPIC-Norfolk cohort [158]. Collectively, these observations pointed out *MARC1* as a potential pharmacologic target for liver diseases without affecting cardiovascular outcomes [234], although further investigations are needed to clarify its function and its role in oxidative stress regulation. A schematic over-view of the main genetic risk factors involved in NAFLD onset and progression is represented in Figure 1 and in Table 1.

## 7. Polygenic Risk Scores (PRSs): From Bench to Bedside and Back

In the last decade, candidate gene studies and GWAS highlighted the impact of single genetic variants on progressive NAFLD. To date, similar to what has been previously carried out for other complex diseases, it is preferred to aggregate individual *loci* into PRSs to estimate the risk to develop severe NAFLD, by using regression models or more complex statistical tools [235]. Then, they performance should be attested by using receiver operating characteristics (ROC) curves and analyzing the area under the curve (AUROC) [236]. The combination of these scores with environmental and dynamic risk factors, considered as covariates, may represent a more appealing approach and may have a greater clinical utility to diagnose those patients at raised risk to progress to severe stages of the disease and to devise effective therapeutic strategies [237].

We have firstly exploited a mendelian randomization analysis and a PRS to prove that fatty liver is the main driver of advanced liver damage and that the effect of *PNPLA3*, *TM6SF2*, *MBOAT7* and *GCKR* at-risk alleles on hepatic injuries is directly proportional to their ability to promote hepatic fat deposition [33]. Likewise, Di Costanzo and colleagues tested the role of metabolic and genetic variables on hepatic fat accumulation in overweight children [238]. They showed that inheritable variations may more tightly participate to fat deposition rather than IR, a well-established trigger of steatosis and fibrosis. Furthermore, hepatic fat content variability was explained for 8.7% by metabolic factors and for 16.1% by inherited *PNPLA3*, *TM6SF2* and *GCKR* variations [238]. A similar approach has been used in a cohort of 2042 pediatric patients by Suomela and collaborators, who confirmed that the combination of genetic and metabolic risk factors, along with BMI, insulin levels and, *PNPLA3* and *TM6SF2* genetic variants, in a predictive score is more trustworthy to foresee fatty liver compared to the one used in adulthood based only on BMI and insulin [239]. Similarly, Koo and colleagues modeled a scoring system, based on genetic and clinical factors, with the pursuit to determine the risk of NASH, in Asian NAFLD patients [240]. In particular, this score which included *PNPLA3* and *TM6SF2* genotypes, IR, diabetes, hepatic enzymes and C-reactive protein and it was able to detect NASH with an AUROC of 0.835 (95% CI, 0.776–0.895) and of 0.809 (95% CI, 0.757–0.861) in NAFLD patients with and without diabetes, respectively.

Moreover, Krawczyk and coworkers investigated the cumulative effect of *PNPLA3*, *TM6SF2* and *MBOAT7* genetic variants on the spectrum of NAFLD and they revealed that the rising number of risk alleles was associated with heavier AST and a trend for increased ALT and γ-glutamyl transferase (GGT) levels, which may mirror the severity of hepatic injury in NAFLD [122]. Additionally, a PRS obtained by considering the number of risk alleles for 6 different SNPs, among which *PNPLA3* rs738409, was strictly correlated with circulating ALT in 178 Mexican NAFLD patients [241]. In a study conducted in 384 Chinese NAFLD patients and 384 age- and gender-matched healthy controls, the number of risk alleles of *PNPLA3* and *TM6SF2* variants strongly correlated with the presence of NAFLD, showing an overall significative odd ratio (OR) of 1.64 which raised in additive manner, with an average increase in OR of 1.52 per additional risk allele [242]. This effect has been even confirmed by Xu and colleagues, which reported an interaction between the PNPLA3 p.I148M and TM6SF2 p.E167K variants in northeast China population, conferring an higher risk to develop NAFLD in patients carrying both mutations (OR: 5.133 in carriers of the p.I148M and p.E167K vs. OR: 1.91 in carriers of the p.I148M variant alone or OR: 3.62 in carriers of the p.E167K variant alone) [243].

In details, the combined effect of *PNPLA3* and *TM6SF2* mutations seems to affect lipid metabolism and NAFLD possibly by exacerbating the expression of genes involved in DNL [244]. The accuracy of PRS to estimate the risk of progressive NAFLD was also evaluated in a Japanese study, in which it has been yielded that the effect of *PNPLA3*, *GATAD2A* and *GCKR* variations was cumulative in the increasing NASH risk, in a dose dependent manner [147].

In addition, we previously reported that the number of heritable risk variants in *PNPLA3*, *TM6SF2* and *MBOAT7* was strongly associated with HCC onset, with a 13.4-fold higher risk in NAFLD patients carrying five risk alleles compared to none [107]. Then, Gellert-Kristensen and colleagues have assessed that a PRS including mutations in *PNPLA3*, *TM6SF2* and *HSD17B13* genes is associated with a 12-fold and 29-fold higher risk to develop cirrhosis and HCC, respectively, as it has been proven by examining 110,761 individuals from the Danish general population, 334,691 individuals from the UK Biobank, and a meta-analysis of the two studies combined [245].

It has been corroborated that inherited variations which influence the susceptibility to hepatic fat content may promote HCC onset and may be worthwhile biomarkers for patients’ stratification. Thus, the impact of each genetic variants on HCC was directly proportional to the predisposition to fatty liver [246]. The utility of a ‘good’ PRS is to constitute a powerful tool to improve the accuracy of HCC detection, predicting HCC more robustly than a single variant and to stratify patients according to the risk of HCC development.

Ultimately, all these studies raised the important question about the possibility to use these scores in the clinical surveillance to predict the development of NAFLD and its progression to more advanced forms and how to combine them with the metabolic risk factors to apply the appropriate pharmacologic and lifestyle interventions.

## 8. Novel Insights into the Modelling of NAFLD: From Genetic Studies to Cellular Models

In the last years, clustered regularly interspaced short palindromic repeats/CRISPR-associated protein 9 (CRISPR/Cas9) became the most broadly used genome editing technique to model NAFLD in vitro and in vivo [247]. For instance, two different *PNPLA3* mutations have been introduced in human cells (*PNPLA3^−/−^* and p.I148M KI cell lines), by using CRISPR/Cas9 by Luukkonen et al. Both cell lines displayed a dramatic intracellular accumulation of LDs, upon unsaturated fatty acids administration, suggesting that PNPLA3 may act as an unsaturated fatty acid-specific hydrolase [62]. The same methodological approach has been exploited by Fan and colleagues to disrupt *TM6SF2* gene in mice. These mice exhibited dampened circulating cholesterol levels, mirroring the phenotype of patients carrying the rs58542926 variant [82]. Similar findings have been observed even in a genetic model of *TM6SF2^−/−^* in zebrafish, in which reduced circulating LDL levels are associated with enhanced hepatic fat deposition [88]. Afterwards, our group generated different HepG2 cell lines carrying the PNPLA3 p.I148M variant and containing the deletion of *MBOAT7*^−/−^ by using CRISPR/Cas9, showing that mutant cell lines acquire the ability to accumulate an increasing number of LDs into the cytoplasm, as a consequence of MBOAT7 downregulation [109]. More recently, we silenced *TM6SF2* in both HepG2 (*TM6SF2^−/−^*) and in *MBOAT7*^−/−^ cells, by CRISPR/Cas9, with the aim to investigate the impact of *TM6SF2* and/or *MBOAT7* depletion on mitochondrial function and morphology. We revealed that both *TM6SF2^−/−^* and *MBOAT7*^−/−/^*TM6SF2^−/−^* are characterized by an enrichment in the number of mitochondria with small and globular shape, loss of cistern architecture and ultrastructural electron density which may indicate mitochondrial failure and degeneration. Notably, the compound KO model runs into metabolic reprogramming towards anaerobic glycolysis, supporting that the co-absence of *TM6SF2* and *MBOAT7* deletions together with the presence of the PNPLA3 p.I148M mutation may synergically affect mitochondrial metabolism within the hepatocytes thus contributing to progressive liver damage and possibly triggers the switch towards HCC [248,249].

In this context, forefront methodological approaches have been exploited to generate induced pluripotent stem cells (iPSCs) from patient-derived fibroblasts or lymphocytes, with the purpose to study the impact of heritable variations in a more reliable situation [250]. Likewise, these cells may be potentially used as a ‘platforms’ to recapitulate patients’ phenotype and to test personalized pharmacological approaches. For instance, Graffmann and colleagues differentiated iPSCs derived from four donors with varying disease stages into hepatocyte-like cells to determine the hepatic metabolic adaptations to oleic acid exposure. These authors highlighted that iPSCs treated with oleic acid can resemble the alterations of glucose and lipid metabolism of the donor. In addition, they stimulated hepatocyte-like cells with a synthetic analogue of adiponectin, in the attempt to reverse steatosis, obtaining variable results [251]. Hepatocyte-like cells have been generated also by Sinton et al. [252], which demonstrated that lactate, pyruvate and octanoate treatment is able to induce macrovesicular steatosis, mitochondrial respiration derangement and electron transport chain dysfunctions, further improving the findings observed by Lyall et al. [253].

## 9. Concluding Remarks

Genetic modifiers exert an essential role in NAFLD pathogenesis and in its evolution towards NASH and HCC. The advantage to address the research to genetic markers investigation is that they do not change during the course of the disease and they may indicate the risk long before clinical symptoms [254]. In the last decades, a growing number of inherited factors has been discovered, albeit the polymorphisms in *PNPLA3*, *TM6SF2* and *MBOAT7* genes are considered the most robust predictors. Nevertheless, many other common variants with modest effect sizes, and various rare variants with small and large effect sizes may participate to NAFLD precipitation towards advanced stages of liver damage. Thus, it remains unclear how interpreter single genetic data and how they can be translated into the clinical context.

To date, it is recommended to combine individual genetic mutations into polygenic risk scores to more accurately figure out the degree of the risk to develop severe NAFLD in patients. Herein, the opportunity to combine polygenic risk scores with routinely assessed biochemical markers may constitute an attractive choice to draft preventive and tailored interventions in subjects at greater risk of aggressive NAFLD.

## Figures and Tables

**Table 1:** Schematic list of the main inherited variations related to the histological hallmarks of NAFLD.

Variant	Gene	Global MAF	Function	Effect	Impact	Phenotype
rs738409 C > G	*PNPLA3*	0.26 (G)	Lipid remodeling	p.I148M	Loss-of-function	↑ NAFLD, NASH, fibrosis, HCC
rs58542926 C > T	*TM6SF2*	0.07 (T)	VLDL secretion	p.E167K	Loss-of-function	↑ NAFLD, NASH, fibrosis
rs641738 C > T	*TMC4/* *MBOAT7*	0.37 (T)	Lipid remodeling	p.G17E	Loss-of-function	↑ NAFLD, NASH, fibrosis, HCC
rs1260326 C > T	*GCKR*	0.29 (T)	Regulation of *DNL*	p.P446L	Loss-of-function	↑ NAFLD, NASH, fibrosis
rs72613567 T > TA	*HSD17B13*	0.18 (TA)	Lipid remodeling	Truncated protein	Loss-of-function	↓ NASH, fibrosis, HCC
rs4841132 G > A	*PPP1R3B*	0.09 (A)	Glycogen synthesis	Non-coding	Gain-of-function	↓ NAFLD, fibrosis, HCC
rs1801278 C > T	*IRS1*	0.05 (T)	Insulin signaling	p.G972R	Loss-of-function	↑ Fibrosis
rs1044498 A > C	*ENPP1*	0.34 (C)	Insulin signaling	p.K121Q	Gain-of-function	↑ Fibrosis
rs2954021 G > A	*TRIB1*	0.45 (A)	Regulation of *DNL*	Non-coding	Gain-of-function	↑ NAFLD
rs12137855 C > T	*LYPLAL1*	0.16 (T)	Lipid metabolism	Intronic	Loss-of-function	↑ NAFLD
Several	*APOB*	NA	VLDL secretion	Protein change	Loss-of-function	↑ NAFLD NASH, fibrosis, HCC
Several	*MTTP*	NA	VLDL secretion	Protein change	Loss-of-function	↑NAFLD
rs236918 G > C	*PCSK7*	0.26 (C)	Membrane transferrin receptor shedding and regulation of circulating lipids	Intronic	Gain-of-function	↑ NASH, fibrosis
Several	*PCSK9*	NA	LDL uptake	Protein change	Loss-of-function	No evidence of association with steatosis
Several	*LIPA*	NA	Lipid remodeling	Protein change	LAL deficiency	↑ NAFLD, NASH, fibrosis
rs56225452 G > A	*FATP5*	0.16 (A)	FFAs uptake	Non-coding	Gain-of-function	↑ NASH, fibrosis
rs13412852 C > T	*LPIN1*	0.21 (T)	Lipid metabolism	Intronic	Not Defined	↓ NASH, fibrosis
rs35568725 A > G	*PLIN2*	0.02 (G)	Lipid remodeling	p.S251P	Loss-of-function	↑ NAFLD, NASH, IR, atherosclerosis
rs884164 A > G	*PLIN5*	0.19 (G)	Lipid remodeling	Non-coding	Loss-of-function	↑ oxidative stress
rs17618244 G > A	*KLB*	0.15 (A)	FGF19/FGFR4 pathway	p.R728Q	Loss-of-function	↓ NASH, fibrosis
rs4374383 G > A	*MERTK*	0.45 (A)	Innate immunity	Intronic	Loss-of-function	↓ Fibrosis
rs3750861 G > A	*KLF6*	0.07 (A)	HSCs activation	Splice variant IVS1-27G	Loss-of-function	↓ Fibrosis
Several	*TERT*	NA	Telomere maintenance	Protein change	Loss-of-function	↑ Fibrosis, HCC
rs12979860 C > T	*IL28B*	0.36 (T)	Innate immunity	Alternative IFNL3/4 transcription	Loss-of-function	↓ NASH, Fibrosis
rs3480 A > G	*FNDC5*	0.42 (G)	HSCs activation	Non-coding	Loss-of-function	↓ Fibrosis
rs4880 C > T	*SOD2*	0.33 (T)	Mitochondrial antioxidant	p.A16V	Loss-of-function	↑ Fibrosis
rs695366 G > A	*UCP2*	0.26 (A)	Mitochondrial lipid metabolism Oxphos	−866 promoter variant	Gain-of-function	↓ NASH, fibrosis
rs2642438 G > A	*MARC1*	0.19 (A)	Mitochondrial detoxification	p.A165T	Loss-of-function	↓ NAFLD, NASH, fibrosis

MAF: minor allele frequency.

## Abbreviations

ACDRP	amish complex disease research program
ALD	alcoholic liver disease
ALT	alanine aminotransferase
APOB	apolipoprotein B
APOC3	apolipoprotein C3
ATGL	adipose triglyceride lipase
AUROC	area under receiver operating characteristics
BMI	body mass index
CCl_4_	carbon tetrachloride CD36: cluster of differentiation 36
CDKI1A	cyclin dependent kinase inhibitor 1A
CGI-58	comparative gene identification-58
CPN1	carboxypeptidase n subunit 1
CRISPR/Cas9	clustered regularly interspaced short palindromic repeats/CRISPR-associated protein 9
CVD	cardiovascular disease
DAGs	diacylglycerols
DNL	*de novo* lipogenesis
ENPP1	ectonucleotide pyrophosphatase/phosphodiesterase1
ER	endothelial reticulum
EWAS	exome wide association studies
FATP5	fatty acid transport proteins
FFAs	free fatty acids
FGF	fibroblast growth factor
FGFR4	fibroblast growth factor receptor 4
FNDC5	fibronectin type III domain-containing protein 5
FOXO1	forkhead box protein O1
GCKR	glucokinase regulator
GGT	γ-glutamyl transferase
GPR55G	protein-coupled receptor 55
GWAS	genome-wide association study
HCC	hepatocellular carcinoma
HFD	high-fat diet
HNRNPUL1	heterogeneous nuclear ribonucleoprotein U like 1
HSCs	hepatic stellate cells
HSD17B13	hydroxysteroid 17-β dehydrogenase 13
IFNL3/4	interferon λ3/λ4
IL28	interleukin 28
InsR	insulin receptor
iPSCs	pluripotent stem cells
IR	insulin resistance
IRAS	Insulin Resistance Atherosclerosis Study
IRS1	insulin receptor substrate
KLB	β-Klotho
KLF6	krueppel-like factor 6
KI	knock-in
KO	knock-out
LAL	lysosomal acid lipase
LD	lipid droplet
LDL	low-density lipoprotein
LIPA	lysosomal acid lipase
LPIAT1	lyso-phosphatidylinositol (Lyso-PI) acyl-transferase1
LPIN1	Lipin1
LPL	lipoprotein lipase
LYPLAL1	lysophospholipase-like 1
MARC1	amidoxime-reducing component 1
MBOAT7	membrane bound o-acyltransferase domain-containing 7
MCD	methionine choline deficient
MERTK	macrophage c-mer tyrosine kinase
MRI-PDFF	magnetic resonance imaging proton-density fat fraction
MTTP	microsomal triglyceride transfer protein
NAD	nicotinamide adenine dinucleotide
NAFLD	nonalcoholic fatty liver disease
NASH	nonalcoholic steatohepatitis
NOMAS	Northern Manhattan Study
OR	odd ratio
PCs	phosphatidylcholines
PCSK7	proprotein convertase subtilisin/kexin type 7
PCSK9	proprotein convertase subtilisin/kexin type 9
PI	phosphatidylinositol
PI3K	Phosphoinositide 3-kinase
PLIN2	perilipin 2
PLIN5	perilipin 5
PNPLA2	patatin-like phospholipase domain-containing 2
PNPLA3	patatin-like phospholipase domain-containing 3
PPP1R3B	protein phosphatase 1 regulatory subunit 3B
PROTAC	proteolysis-targeting chimera
PRSs	polygenic risk scores
PS	phosphatidylserine
PUFAs	polyunsaturated fatty acids
ROC	receiver operating characteristics
ROS	reactive oxygen species
SIRTs	sirtuins
SNP	single nucleotide polymorphism
SOD2	superoxide dismutase 2
SREBP1c	sterol regulatory element-binding protein 1
T2D	type 2 diabetes
TERT	telomerase reverse transcriptase
TG	triglyceride
TGF-β	transforming growth factor β
TLR4	toll like receptor 4
TM6SF2	transmembrane 6 superfamily member 2
TMC4	transmembrane channel like 4
TNF-α	tumor necrosis factor α
TRIB	tribbles homolog1
UCP	uncoupling protein
VLDL	very-low density lipoproteins
